# The Prevalence of Undiagnosed HIV Infection in Those Who Decline HIV Screening in an Urban Emergency Department

**DOI:** 10.1155/2011/879065

**Published:** 2011-05-09

**Authors:** M. Czarnogorski, J. Brown, V. Lee, J. Oben, I. Kuo, R. Stern, G. Simon

**Affiliations:** ^1^Division of Infectious Diseases, Department of Medicine, The George Washington University, Washington, DC 20037, USA; ^2^Department of Emergency Medicine, The George Washington Medical Center, Washington, DC 20037, USA; ^3^Department of Epidemiology and Biostatistics, The George Washington University, Washington, DC 20037, USA

## Abstract

*Objective*. To determine the prevalence of occult HIV infection in patients who decline routine HIV testing in an urban emergency department. *Design, Setting, and Patients*. Discarded blood samples were obtained from patients who had declined routine ED HIV testing. After insuring that the samples came from patients not known to be HIV positive, they were deidentified, and rapid HIV testing was preformed using 5 *μ*L of whole blood. *Main Outcome Measures*. The prevalence of occult HIV infection in those who declined testing compared with prevalence in those who accepted testing. *Results*. 600 consecutive samples of patients who declined routine HIV screening were screened for HIV. Twelve (2%) were reactive. Over the same period of time, 4845 patients accepted routine HIV testing. Of these, 35 (0.7%) were reactive. The difference in the prevalence of HIV infection between those who declined and those who accepted testing was significant (*P* = .001). The relative risk of undetected HIV infection in the group that declined testing was 2.74 times higher (95% CI 1.44–5.18) compared with those accepted testing. *Conclusion*. The rate of occult HIV infection is nearly three-times higher in those who decline routine ED HIV testing compared with those who accept such testing. Interventions are urgently needed to decrease the opt-out rate in routine ED HIV testing settings.

## 1. Introduction

The Centers for Disease Control and Prevention (CDC) has estimated that approximately 1–1.2 million people in the US are infected with HIV. Of these, one-quarter are unaware of their infection [1, 2]. In 2006, the CDC recommended that routine HIV screening be expanded to many outpatient settings including emergency departments (EDs). Specifically, the CDC recommended that screening for HIV infection be routinely performed for all patients aged 13–64, in settings where the prevalence of undiagnosed HIV infection in the patient population is more than 0.1% [3]. The CDC also recommended that testing programs use an opt-out approach in which patients are informed that the test will be done unless they decline. Several emergency departments have instituted these recommendations, including The George Washington University Hospital (GWUH), which began to offer routine opt-out ED HIV testing in September 2006 [4, 5]. Since the program's inception, the GWUH-ED has offered free rapid HIV testing to over 32,000 individuals. Approximately 44% of eligible patients decline an ED HIV test, and the HIV seroprevalence rate among those who accepted testing is 0.7% [4, 6]. Our prior work demonstrated that the main reason patients declined routine ED HIV screening is that they did not believe they were at risk for HIV infection [7]. We hypothesized that patients underestimate their HIV risks, and that the rate of undetected HIV in patients who decline testing would be as high as the rate in those who accept the test. We, therefore, performed a study to compare the HIV seroprevalence rate of individuals who declined routine ED testing with that of patients who accepted testing. 

## 2. Materials and Methods

This cross-sectional study was performed at The George Washington University Hospital Emergency Department in Washington, DC. The George Washington University Hospital is a 370-bed, urban, tertiary care center and a level-1 trauma center located in Washington DC where the HIV seroprevalence is approximately 3% [8]. The ED census is 62,000 patient visits per year. In 2006, the GWU ED implemented an opt-out, nontargeted HIV screening program in response to CDC guidelines on non-targeted HIV screening. Characteristics of this program have been described in detail elsewhere [4, 6]. In brief, a patient was eligible for ED HIV screening if he or she was aged 18–64, was not known to be HIV positive, was able to communicate with the screener and had a normal mental status, had not been tested within the prior three months, and had no urgent medical condition that required immediate intervention. HIV screening was performed by dedicated additional screening staff available 24 hours a day, using the OraQuick Advance Rapid HIV-1/2 Antibody Test (OraSure Technologies, Inc, Bethlehem, PA). The type of HIV screening test made available for clinical use was both determined and provided by the HIV/AIDS, Hepatitis, STD, and TB Administration in the DC Department of Health. The screeners reviewed the ED electronic medical record for eligible patients. Once a patient was determined to be eligible for a test, he or she was informed by the screeners that they would be tested unless they declined the test. There was no pretest counseling, and written informed consent was not required. Preliminary HIV test results were recorded in the patient's electronic medical record in the ED. Western blot confirmatory testing was conducted for all preliminary positives among those who accepted screening. The prevalence of HIV infection in those who accepted the test was calculated as the number of confirmed positive tests divided by the number of unique patients who accepted screening during the study period.

To estimate the HIV prevalence among emergency department patients who declined HIV screening, discarded blood samples were collected daily between July 26, 2008 and March 26, 2009. These samples had been collected for diagnostic testing but were no longer needed. The discarded samples were included in this analysis if they met the following criteria: (1) they belonged to a patient who was offered and refused HIV screening in the ED and (2) the sample was collected in an appropriate container (lithium heparin, sodium citrate, or EDTA). To insure that these samples did not contain patients who were known to be HIV positive, two steps were taken. Firstly, the name on each sample was checked against a database of patients who had previously been offered a routine HIV screening test. If the patient had taken the test and was positive, the sample was excluded. Secondly, the name was checked against all prior ED visits (going back to 2004) for evidence of known HIV infection, such as a report of the disease in the patient's medical history, a positive confirmatory test for HIV, or the presence of any antiretroviral medication in the list of the patient's medications. The sample was excluded if there were any of these findings. 

Demographic data for patients who accepted or declined the test were obtained from the ED electronic medical record. In addition, the patient's reason for declining testing was recorded. After this initial data was collected, the identifying patient label was removed from the blood sample, and identifying information was deleted from the electronic database. The sample and the de-identified database entry were then linked with a unique study identification number. HIV screening was performed with 5 *μ*L of whole blood using the OraQuick Advance Rapid HIV-1/2 Antibody Test, the same test that was used for patients who accepted screening.

All patients signed a consent form permitting de-identified discarded blood samples to be used for scientific purposes, and the study was approved by the Institutional Review Board at the George Washington University Medical Center.

The sample size calculation was based on assumptions of the expected HIV prevalence in both groups. With *α* = 0.05 and a power of 0.8, 590 samples would be needed if the prevalence was 1% in the patients who accepted testing and 3% in those who declined, assuming at least 2,500 patients were in the group that accepted testing. Demographic characteristics, including age, sex, race, and insurance status of the acceptors and the decliners were compared using Chi-square tests. 

## 3. Results

**Figure 1:**
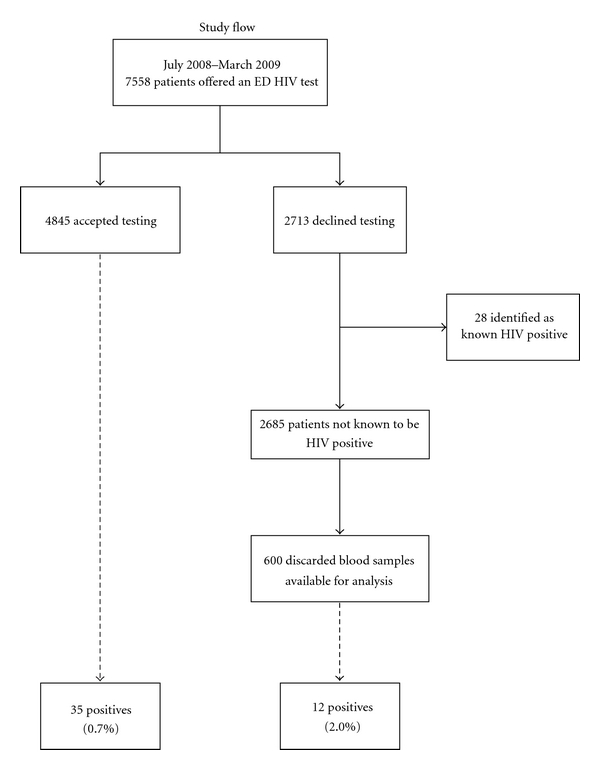
Study flow.

During the study period, 7,558 patients met eligibility criteria for routine screening, of whom 4,845 (64%) accepted testing and 2,713 individuals declined. Of those who declined testing, we identified 28 patients who were already known to be HIV positive. From the remaining 2,685 patients, we screened 600 consecutive patients who also had a discarded blood sample (see Figure 1). The demographic characteristics of all individuals offered HIV testing in the ED are presented in Table 1. There were no significant differences in age, race, or insurance status between those who agreed to be tested and those who declined. 

Of the 600 samples analyzed, 12 (2%) were reactive, compared with 35 (0.7%) reactive samples in the group accepting HIV screening during the same period (*P* = .001). The relative risk of occult HIV infection in the group that declined testing was 2.74 (95% CI 1.44–5.18) compared to the group that accepted testing (see Table 1). There was a significant difference in the genders of those who were found to be HIV positive between the two groups. In the group that accepted HIV screening, 20% (7/35) of the seropositive individuals were women; in the group that declined testing, 67% (8/12) of those who were seropositive were women (*P* < .05, see Table 2). Being nonwhite was associated with declining testing and having a positive HIV test (Table 3). 

The most common reason for declining testing was the patient's belief he or she was not at risk. Of the 600 samples from patients who declined testing, 49% stated the reason for not testing was “I am not at risk” (Table 2). Among those who declined testing and had a positive screen, one-third (4/12) stated that they were not at risk (Table 2). 

## 4. Discussion

In this ED HIV screening program, patients who declined testing had a positivity rate of 2%, almost three-times the rate of those who accepted the test during the same time period. 

Although we believe ours is the first study of its type, data from women who opt out of prenatal HIV screening supports our conclusions. In one study, between 3.6 and 4.3% of pregnant women declined an HIV screening test, and the seroprevalence of HIV in this group was found to be 3.3-times higher than the HIV seroprevalence among pregnant women who accepted the screening test [9]. Individuals who decline a routine HIV screening and who may assume that they have little risk of HIV infection appear to be at considerably greater risk than they believe. 

There are several explanations for our finding. It is possible that some of the patients who declined testing actually knew they were HIV positive, but were uncomfortable in reporting this information. For this reason, they stated they were not at risk when asked why they declined a test. While we cannot completely exclude this possibility, we believe it is unlikely. ED patients are always asked about any underlying medical conditions, and for a list of the medications that they are taking. This information is gathered by the nurse at triage, and is reviewed by the treating physician who asks again about prior medical conditions and medications. These details are recorded on the ED electronic medical record, and in our experience patients are generally forthcoming about their medical history, including a history of HIV infection. We attempted to minimize the possibility of including a patient who had not shared their HIV-positive status by reviewing all the available medical records for any reference to HIV infection, before determining that a patient was not known to be HIV positive. 

Another possibility is that patients who declined a screening test were actually aware of their own increased risk of HIV infection, but did not wish be tested for fear of learning their status. These patients, therefore, declined testing, and offered a reason that they felt to be the most socially acceptable. This phenomenon, known as social desirability bias, is well described in the literature and may apply to this cohort of patients [10]. 

Another consideration is that patients who are more seriously ill are more likely to have blood drawn during their ED visit. This would lead to sicker patients being overrepresented in the discarded blood samples when compared with the cohort of patients who accept an HIV screening test. Since the entire cohort of patients we tested did have blood drawn, they may as a whole have been sicker than patients in the comparator group. Since we did not record which patients in the comparator group had blood drawn, we cannot evaluate this possibility further. 

Many HIV-infected persons learn about their diagnosis years after initial infection [11]. Missed opportunities for HIV testing occur in medical settings frequently, and it has been shown that there is a high rate of undiagnosed HIV infection among the patient population seen in urban emergency departments [12, 13]. The 2006 CDC recommendations aimed to increase routine HIV testing in medical care to help identify previously undiagnosed HIV infection earlier in their disease. As these recommendations have slowly been implemented, it has been noted that the incidence of HIV infection is highest among racial and ethnic minorities who have poor access to healthcare and frequently utilize the ED as their predominant source of health care [12]. Most of these patients with undiagnosed HIV infection, present to the ED for reasons unrelated to their infection. As a result, it is precisely those patients who are at a greater risk of infection who are most likely to go undetected early in the course of their HIV disease [14]. 

Our finding of a high rate of HIV infection among women who decline testing is of great concern. These women, by virtue of their belief that they are not infected may be more likely to engage in additional risky behavior and contribute to the spread of the HIV virus. Women in particular appear to be at increased risk for having HIV infection yet underestimate their risk [15]. These findings parallel the recent assessment of the changing face of the HIV epidemic in the District of Columbia conducted by DC DOH and George Washington School of Public Health [16]. Although blacks, other nonwhites, and patients under the age of 50 had high rates of unrecognized infection and underestimated risk, the greatest increase in incidence was among African-American women. 

In conclusion, this study demonstrated that patients who decline routine HIV screening in an emergency department in a high prevalence area are at a higher risk of infection compared with those who accept testing. Although routine HIV testing in the ED has been shown to be an effective strategy at identifying HIV infections in the community [6, 12, 17, 18], screening ultimately depends on the patient's willingness to accept the test. We suggest that interventions be targeted at those who decline routine testing and especially to black women, who may be significantly underestimating their risk of infection. 

## Figures and Tables

**Table 1:** Demographic characteristics and HIV seroprevalence among HIV test acceptors and decliners.

Characteristic	Total (%)	Acceptors (%)	Decliners (%)	*X* ^2^
	*N* = 5445	*n* = 4845	*n* = 600	*P* value
Age				
<35 years old*	3040 (55.8)	2784 (57.5)	256 (42.6)	<.001
≥35 years old	2405 (44.2)	2061 (42.5)	344 (57.4)	

Gender				
Male*	2182 (40.1)	1969 (40.6)	215 (35.7)	.07
Female	3262 (59.9)	2875 (59.4)	385 (64.3)	
Transgender	1 (0.0)	1 (0.0)	0 (0.0)	

Race				
White*	1810 (33.3)	1566 (32.4)	244 (40.7)	<.001
Black	3089 (56.9)	2794 (57.8)	295 (49.2)	
Asian	75 (1.4)	62 (1.3)	13 (2.2)	
Other	278 (5.1)	253 (5.2)	25 (4.2)	
Do not know/unsure	193 (3.3)	170 (3.3)	23 (3.7)	

Insurance				
Private*	2812 (51.8)	2694 (55.7)	118 (19.7)	<.001
Public	735 (13.5)	340 (7.0)	395 (65.9)	
None/self-pay	506 (9.3)	492 (10.2)	14 (2.3)	
Unknown/other	1392 (25.4)	1319 (27.1)	73 (12.0)	

HIV test result				
Positive	47 (0.9)	35 (0.7)	12 (2.0)	.001

*Comparison used to determine *P* value.

**Table 2:** Reason for declining an HIV test.

	Total	Age		Race*		Gender	
Reason	*N* = 600 %	<35 years *n* = 256 %	≥35 years *n* = 344 %	White *n* = 244 %	Non-white *n* = 356 %	Male *n* = 215 %	Female *n* = 385 %

Not at risk	49.2	54.5	45.5	40.3	62.3	46.3	50.9
Recently tested (but more than 3 months prior)	14.2	12.6	15.5	17.5	9.4	13.6	14.6
Would rather be tested somewhere else	3.0	2.8	3.2	3.1	2.9	2.3	3.4
Afraid to get result	2.3	3.1	1.8	3.4	0.8	2.8	2.1
No time in the ED	0.8	0.4	0.9	0.9	0.8	0.9	0.8
No reason given	26.7	22.8	29.7	31.3	20.1	30.8	24.4
Other	3.7	3.9	3.5	3.7	3.7	3.3	3.9

*P < .001.

**Table 3:** HIV seropositivity among decliners by age, race, gender, and main reasons for declining (*n* = 600).

Characteristic	HIV negative *n* = 588	HIV positive *n* = 12	*P*-value*
Age			
<35 years old	253 (98.4)	4 (1.6)	.57
≥35 years old	335 (97.7)	8 (2.3)	
Race			
White	244 (99.6)	1 (0.4)	**.03**
Non-white	344 (96.9)	11 (3.1)	
Gender			
Male	211 (98.1)	4 (1.9)	1.00
Female	377 (97.9)	8 (2.1)	
Felt was not at risk for HIV			
Yes	297 (97.4)	8 (2.6)	.38
No	291 (98.6)	4 (1.4)	
Recently tested			
Yes	505 (98.1)	10 (1.9)	.68
No	83 (97.7)	2 (2.3)	
Would rather test somewhere else			
Yes	296 (97.4)	8 (2.6)	.31
No	292 (98.6)	4 (1.4)	

*Fisher's exact test due to cell sizes <5.

## References

[1] Campsmith ML, Rhodes P, Hall HI, Green T (2008). HIV prevalence estimates—United States, 2006. *Morbidity and Mortality Weekly Report*.

[2] McQuillan G, Kruszon-Moran D HIV Infection in the United States Household Population Aged 18–49 Years: results from 1999–2006. http://www.cdc.gov/nchs/data/databriefs/db04.pdf.

[3] Branson BM, Handsfield HH, Lampe MA (2006). Revised recommendations for HIV testing of adults, adolescents, and pregnant women in health-care settings. *Morbidity and Mortality Weekly Report. Recommendations and Reports*.

[4] Brown J, Shesser R, Simon G (2007). Routine HIV screening in the emergency department using the new US centers for disease control and prevention guidelines: results from a high-prevalence area. *Journal of Acquired Immune Deficiency Syndromes*.

[5] Brown J, Shesser R, Simon G (2007). Establishing an ED HIV screening program: lessons from the front lines. *Academic Emergency Medicine*.

[6] Brown J, Magnus M, Czarnogosrki M, Lee V (2010). Another look at Emergency Department HIV screening in practice: no need to revise expectations. *AIDS Research and Therapy*.

[7] Brown J, Kuo I, Bellows J (2008). Patient perceptions and acceptance of routine emergency department HIV testing. *Public Health Reports*.

[8] Annual Report (2009). HIV/AIDS, Hepatitis, STD and TB Epidemiology.

[9] Plitt SS, Singh AE, Lee BE, Preiksaitis JK (2007). HIV seroprevalence among women opting out of prenatal HIV screening in Alberta, Canada: 2002–2004. *Clinical Infectious Diseases*.

[10] Crowne DP, Marlowe D (1960). A new scale of social desirability independent of psychopathology. *Journal of Consulting Psychology*.

[11] CDC (2003). Late versus early testing of HIV—16 Sites, United States, 2000-2003. *Morbidity and Mortality Weekly Report*.

[12] Calderon Y, Leider J, Hailpern S (2009). High-volume rapid HIV testing in an Urban emergency department. *AIDS Patient Care and STDs*.

[13] Kelen GD, Hexter DA, Hansen KN, Tang N, Pretorius S, Quinn TC (1995). Trends in human immunodeficiency virus (HIV) infection among a patient population of an inner-city emergency department: implications for emergency department-based screening programs for HIV infection. *Clinical Infectious Diseases*.

[14] Johnson DF, Sorvillo FJ, Wohl AR (2003). Frequent failed early HIV detection in a high prevalence area: implications for prevention. *AIDS Patient Care and STDs*.

[15] Stevens P, Keigher S (2009). Systemic barriers to health care access for U.S. women with HIV: the role of cost and insurance. *International Journal of Health Services*.

[16] District of Columbia HIV/AIDS Epidemiology Update 2008.

[17] Kelen GD, Rothman RE (2009). Emergency department-based HIV testing: too little, but not too late. *Annals of Emergency Medicine*.

[18] White DAE, Scribner AN, Schulden JD, Branson BM, Heffelfinger JD (2009). Results of a Rapid HIV Screening and Diagnostic Testing Program in an Urban Emergency Department. *Annals of Emergency Medicine*.

